# Exploring the Potential of Duck Egg White Jelly: Enhancing Texture, Reducing Phosphate, and Innovating Emulsified Meat Snacks

**DOI:** 10.3390/foods13233892

**Published:** 2024-12-02

**Authors:** Nian-Yao Zheng, Yen-Po Chen, Yu-Cheng Liu, Jia-Shian Shiu, Lian-Ben Chang, Sheng-Yao Wang

**Affiliations:** 1 Department of Animal Science and Technology, National Taiwan University, Taipei 10617, Taiwan; ben2002204@gmail.com (N.-Y.Z.); xu6m4tp2012@gmail.com (Y.-C.L.); d01626003@ntu.edu.tw (L.-B.C.); 2 Department of Animal Science, National Chung Hsing University, Taichung 40227, Taiwan; chenyp@dragon.nchu.edu.tw; 3 The iEGG and Animal Biotechnology Center, National Chung Hsing University, Taichung 40227, Taiwan; 4 Southern Region Branch, Taiwan Livestock Research Institute, Ministry of Agriculture, Pingtung 946, Taiwan; mucleshank12@gmail.com

**Keywords:** duck egg white jelly, emulsified meat snack, phosphate, texture

## Abstract

Duck egg white jelly, a protein-rich, alkali-induced gel, mirrors preserved duck egg white in appearance and properties, offering easier storage and utility, especially when excess egg white is available. This research focuses on incorporating duck egg white jelly into emulsified meat snacks to enhance texture while reducing the phosphate content. This study suggests that adding phosphate and duck egg white jelly increases raw meat paste pH, affecting its viscosity. With half the usual phosphate and either 3.0% or 6.0% jelly, the pH significantly increases compared to the control paste, containing 0.2% phosphate (*p* < 0.05). Viscosity remains unaffected in the group with 6.0% jelly and no phosphate versus the control (*p* > 0.05). The least favorable viscosity is observed in pastes without phosphate or jelly, suggesting that the jelly plays a similar role to phosphate. After boiling and shaping the pastes into emulsified meat snacks, their texture profiles and water-holding capacities were analyzed. Formulas with phosphate and jelly produced emulsified meat snacks with improved springiness, chewiness, reduced cooking loss, and decreased purge loss during storage. Color analysis showed no significant differences between the control and treatment groups (*p* > 0.05). Duck egg white jelly, when added, effectively reduces the phosphate content while enhancing texture and consumer acceptance of emulsified meat snacks. It serves as a versatile ingredient for low-phosphate, emulsified meat products, with potential for various meat combinations.

## 1. Introduction

Emulsified meatballs have gained popularity in Taiwan and among various ethnic Chinese communities. Traditionally, pork legs were pounded with wooden sticks or stones until they transformed into a raw meat paste. The paste was then manually squeezed into spherical meatballs and boiled to solidify their shape [1]. Alternatively, raw meat pastes can be shaped by hand into various forms and boiled to fix their shape, or squeezed into sausage casings, boiled, and cut into cylindrical pieces that resemble emulsified meatballs. These products, which we term “emulsified meat snacks”, offer a flexible variation on traditional meatballs. Livestock leg meat and poultry breast meat, which are leaner cuts, are commonly used to produce various emulsified meat products, especially those resembling meatballs. The raw lean meat is mechanically broken down, and salts are added to extract salt-soluble proteins. These proteins then attach to the surface of the fat and form a stable meat emulsion with other crushed ingredients [2,3]. These proteins become denatured and set upon boiling, forming a thermally irreversible, stable, and flexible emulsified meat product [4].

To improve the emulsification stability of raw meat pastes and enhance the quality of emulsified meat products, it is essential to incorporate phosphates within a targeted range of 0.10% to 0.20%. Phosphate effectively increases protein solubility in lean meat, more so than salt, by raising the pH and ionic strength of raw meat pastes, without inducing excessive saltiness or dehydration [5,6,7]. This pH shift promotes protein unfolding by increasing electrostatic repulsion when the pH exceeds the isoelectric point of primary meat proteins, a mechanism that significantly improves water-holding capacity, protein dispersion, and water retention in the final product [7,8]. Furthermore, phosphates act as polyelectrolytes in the aqueous phase, enhancing ionic strength and electrostatic repulsion among proteins, thereby creating additional space for bound water. This improves water retention, reduces drip and cooking losses, and enhances the sensory qualities and economic value of emulsified meat products [9,10].

Growing consumer demand for simpler, additive-free food formulations has driven the ‘clean label’ movement, emphasizing essential additives, natural ingredients, and transparency [11]. Phosphates are commonly used in emulsified meat products for their unique ability to enhance quality. However, recent EFSA guidelines limit phosphate use in processed meats to 5000 mg/kg (as P_2_O_5_), prompting the search for effective alternatives [12]. Alkaline additives such as eggshell and oyster shell powders show potential for increasing pH and water retention while reducing cooking loss [13,14]. Zhou et al. [15] found that partially replacing phosphates with sodium bicarbonate improves water retention, gel strength, and texture in mixed meats, allowing for reduced phosphate use. Wang et al. [16] demonstrated that chickpea protein isolate and chitosan can enhance emulsion stability, hardness, and chewiness, positioning them as promising phosphate substitutes. Yuan et al. [17] also reported that seaweed dietary fiber improves cooking yield, texture, and pH in phosphate-free frankfurters due to its high water absorption and mild alkalinity. Other polysaccharides, such as guar gum, carrageenan, and konjac, have also shown promise, improving viscosity and water retention, although they affect texture and sensory properties differently from phosphates [18,19,20,21]. While these alternatives can stabilize meat emulsions, they may reduce hardness, cohesiveness, and chewiness, highlighting the need for further development in phosphate-free formulations [22].

Egg white, known for its excellent gelling and emulsification properties, is rich in proteins, with optimal compositions and ratios [23]. Adding egg white powder to lean meat and fat during processing enhances the quality, texture, and sensory attributes of emulsified meat products while boosting their nutritional value. This combination also strengthens the protein gel network, potentially reducing the need for phosphates [24,25]. Although duck egg white shares similar properties with chicken egg white, its strong gamey flavor and limited acceptance restrict its use. In salted egg production, duck egg yolks are often separated and cured with salt and maltodextrin, leaving substantial amounts of duck egg white as an agricultural by-product [26]. Utilizing surplus duck egg whites in emulsified meat products could support agricultural resource recycling and contribute to a circular economy.

However, duck egg white has a liquid consistency, which can result in issues such as foaming or uneven pastes when mixed with minced meat. Using heat-induced duck egg gel does not enhance the emulsifying stability of meat pastes; instead, it disrupts the network structure during mixing and boiling. To address this, Zheng et al. [27] employed an alkaline treatment technique to produce translucent, heat-stable duck egg white jelly from fresh duck egg white. They successfully developed an alkaline protein-rich gel that maintained its integrity even when subjected to heat, avoiding liquefaction. The jelly exhibited characteristics similar to preserved duck egg white, such as translucence and flexibility. The unfolding protein’s side-chain groups could potentially form bonds or interact with water molecules or protein groups present in the meat. However, no studies have explored the utilization of alkali-induced egg white jelly in emulsified meat products. Therefore, the current study aims to investigate the effects of duck egg white jelly on the quality of raw meat pastes and emulsified meat products. The potential of duck egg white jelly as a substitute for phosphate will also be evaluated. This study addresses the issue of duck egg white wastage and contributes to the reduction of phosphate additives in emulsified meat products.

## 2. Materials and Methods

### 2.1. Materials

Fresh duck eggs (approximately 70 g each) were purchased from Kindly Eggs in Pingtung, Taiwan, and kept at room temperature. They were utilized within one week of being laid. The frozen lean pork meat (back legs) and back fat were purchased from Shang Lee Food in Nantou, Taiwan. All chemicals used in this study were of analytical or food grade. Phosphate and condiments were purchased from Chien Yuan in Taipei, Taiwan. The NOJAX 29/70 cellulose casings were purchased from Viskase in Lombard, IL, USA.

### 2.2. Preparation of Duck Egg White Jelly

Duck egg white jelly was prepared following the procedure described by Zheng et al. [27], with minor modifications. Fresh duck eggs were cracked manually, and the egg whites were carefully separated and collected in a beaker. A 0.6 M sodium hydroxide (NaOH) solution was added to the egg whites at a volume ratio of 4:1 (duck egg white to 0.6 M NaOH solution) while stirring with an electric stirrer at 300 rpm for 30 s, resulting in a final NaOH concentration of 0.15 M in the mixture. The mixture was then allowed to stand at 25 °C for 60 min, forming a jelly-like consistency through alkali-induced gelation. The final pH of the jelly was approximately 11.2. The jelly was then stored at 4 °C until further use.

### 2.3. Preparation of Emulsified Meat Snacks

In this study, lean pork meat from the hind leg of pigs and back fat were mixed in a 3:1 ratio to prepare emulsified meat snacks. The percentage of seasonings and additives in the formulations was determined on the basis of the total combined weight of the lean pork meat and back fat. The selection of hind leg pork ensured a lean meat source with low fat content, contributing to the consistent quality and texture of the emulsified meat snacks. The experimental treatments were categorized into three main groups, based on phosphate addition: group A, which was phosphate-free, group B, with 0.1% phosphate added, and group C, with 0.2% phosphate added. Within these three main groups, further subdivisions were defined on the basis of the addition of 1.5%, 3.0%, and 6.0% duck egg white jelly in the formulations. These groups were labeled with superscripts indicating the percentage of duck egg white jelly added, including A^1.5^, A^3^, A^6^, B^1.5^, B^3^, B^6^, C^1.5^, C^3^, and C^6^ groups. The A^0^ group served as the negative control, with no addition of phosphate or duck egg white jelly. The control group (C^0^; CON) adhered to a commercial meatball formula, with 0.2% phosphate and no duck egg white jelly added. The detailed formulations of the emulsified meat snacks are summarized in Table 1.

Emulsified meat snacks were prepared using a commercial-scale technique proposed by Ding et al. [28], with some modifications. Briefly, thawed lean pork meat (stored at approximately −5 °C) was minced for 10 s using a bowl cutter (K3 Slicer; Kinn Shang Hoo Iron Works, Kaohsiung, Taiwan). Salt (1.3%, *w*/*w*) and phosphate (0%, 1%, and 2%, *w*/*w*) were then incorporated into the minced lean meat, which was processed for approximately 2 min to attain a cohesive texture. Subsequently, ground back fat, egg white jelly (0%, 1.5%, 3%, and 6%, *w*/*w*), and seasonings, including sugar (2.0%, *w*/*w*), garlic powder (0.2%, *w*/*w*), and white pepper powder (0.2%, *w*/*w*), were added and mixed for 3 min to form raw emulsified meat pastes. All materials and raw meat pastes were maintained below 10 °C throughout the processing. The raw meat pastes were then filled into cellulose casings with a diameter of 26 mm and cooked in water at 80 °C for 15 min. After cooling, the cooked samples were removed from their casings and cut into uniform cylinders measuring 26 mm, resulting in emulsified meat snacks. Finally, the samples were vacuum-packed in high-density polyethylene (HDPE) bags (Taipei Pack Industries, Taipei, Taiwan) and stored at 4 °C or −20 °C for subsequent analysis.

### 2.4. Measurement of pH and Viscosity of Raw Emulsified Meat Pastes

The pH of raw emulsified meat pastes was determined in triplicate using a pH meter (Twinno PH30; Strider Technologies, New Taipei, Taiwan) with a penetration probe applied to different sample areas. The viscosity of the pastes was measured using a rheometer (RST-CPS; Brookfield Engineering Laboratories, Middleborough, MA, USA). A constant shear rate of 60 s^−1^ was achieved using a plate–plate geometry with 50 mm diameter plates (RPT-50) separated by a 1 mm gap. For each experimental group, viscosity measurements were performed in triplicate to obtain a representative mean value. A sample of raw emulsified meat paste was then placed on the testing plate, and the sample’s viscosity was measured at a shear rate of 60 s^−1^. Observations were recorded every 10 s at 4 °C for each experimental group to monitor viscosity fluctuations. The mean viscosity was calculated from these triplicate readings, with results expressed as mean ± standard error of the mean (SEM).

### 2.5. Texture Profile Analysis

A TA.XTplus Texture Analyzer (Stable Micro Systems, Godalming, UK) was employed to analyze the texture profile of emulsified meat snacks prepared in three independent batches, each with three replicate samples. For each batch, three cylindrical samples with a diameter of 26 mm and a height of 10 mm were prepared. The analysis was conducted at room temperature (25 °C). Each sample was subjected to two compressions using a P/50 cylindrical probe (aluminum cylinder, 50 mm diameter; Stable Micro Systems), compressing each sample to 60% of its original height at a test speed of 5 mm/s. The mean values of hardness (N), springiness (dimensionless), cohesiveness (dimensionless), chewiness (N·mm), and gumminess (N) were calculated from these three replicate measurements per batch.

### 2.6. Cooking Loss

The cooking loss was assessed using a modified version of the method proposed by Wu et al. [29]. Initially, 100 g of raw emulsified meat paste was vacuum-sealed in an HDPE bag and heated at 80 °C for 15 min until the core temperature reached 80 °C. Afterward, the cooked sample was gently blotted and weighed once its core temperature was reduced to approximately 25 °C by immersion in cold water. The cooking loss was expressed as a percentage using the following formula: cooking loss (%) = (raw weight − cooked weight)/(raw weight) × 100.

### 2.7. Purge Loss

The purge loss of the meat snacks from different groups was evaluated using a method proposed by Ding et al. [28] with some modifications. Initially, the meat snacks were gently wiped with filter paper to remove superficial moisture and weighed (*W*_i_) before being vacuum-packed in HDPE bags. The bags were then stored at −20 °C for 0, 7, 14, 21, and 28 days. Subsequently, the meat snacks were thawed at 4 °C for 8 h, and the purge loss was measured. After removing the meat snacks from the bags, any condensation on the surface of the meat snacks was wiped off with filter paper, and the meat snacks were weighed again (*W*_t_). The purge loss was expressed as a percentage using the formula: purge loss (%) = (*W*_i_ − *W*_t_)/*W*_i_ × 100.

### 2.8. Color Measurement

The surface colors of the meat snack samples obtained using different formulas were measured using a color checker (Model NR-11; Nippon Denshoku Industries, Tokyo, Japan), and the CIE-L*, a*, and b* parameters (lightness, L*; redness, a*; and yellowness, b*) were determined. The meat snack samples were then cut into cylinders with a thickness of 10 mm using a knife for color measurement. After black-and-white calibration, the surfaces of the cylinders were directly analyzed using the color checker, taking into account the L*, a*, and b* values. The sample whiteness was calculated as follows:

$$Whiteness=100-\sqrt{(100-L^{*}{)}^{2}+a^{*2}+b^{*2}}$$

### 2.9. Sensory Evaluation

A sensory evaluation was conducted to assess the sensory attributes of 11 types of emulsified meat snacks using a 9-point hedonic scale. The evaluation involved 35 semi-trained panelists from the Department of Animal Science and Technology at National Taiwan University, including students and faculty members familiar with meat products (19 men [54.29%] and 16 women [45.71%], aged 20–31 years). The panelists were considered semi-trained as they received an introduction to sensory evaluation principles before the test, including instructions on how to use the 9-point hedonic scale and evaluate attributes such as color, texture, flavor, and overall quality. Additionally, their academic and professional familiarity with meat products provided them with foundational knowledge relevant to the evaluation process. Before the evaluation, panelists were informed about the principles of sensory evaluation and provided informed consent to participate. They were also notified about potential allergens in the samples, including duck egg white and pork, to ensure their safety. Each type of meat snack was heated in boiling water for 10 min, placed on 6-inch white paper plates labeled with unique three-digit codes, and presented to panelists in random order at room temperature. Using a 9-point hedonic scale (where 1 = strongly dislike and 9 = strongly like), the panelists evaluated each sample based on color, texture, flavor, and overall quality.

### 2.10. Statistical Analysis

Each treatment condition was replicated three times to ensure the robustness of the results. All collected data were analyzed by SAS 9.4 (SAS Institute Inc., Cary, NC, USA) and presented by average ± standard error of mean (SEM). A one-way analysis of variance (ANOVA), followed by Fisher’s least significant difference (LSD), was conducted at a 95% confidence level to determine the effects of duck egg white jelly.

## 3. Results and Discussion

### 3.1. Effects of Alkaline Duck Egg White Jelly on the pH and Viscosity of Raw Meat Pastes

**Figure 1:**
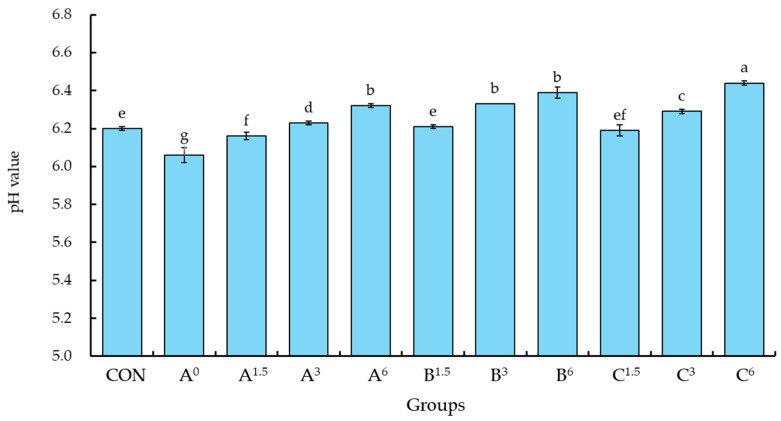
Effects of duck egg white jelly on the pH of raw meat pastes. Raw meat pastes were prepared with three treatments: A (no phosphate), B (0.1% phosphate), and C (0.2% phosphate). The superscript numbers denote the percentage of duck egg white jelly added. Values are presented as means ± SEM (*n* = 3). Bars with different letters indicate significant differences among groups (*p* < 0.05). CON (control) represents a commercial meatball formula containing 0.2% phosphate without duck egg white jelly.

The effects of incorporating duck egg white jelly on the pH and viscosity of raw meat pastes are presented in Figure 1. The pH of the raw meat paste in the control group reached approximately 6.2, which was significantly higher than that of the group A^0^, which did not include phosphates or duck egg white jelly (*p* < 0.05). When the raw meat paste preparation process included only 0.1% phosphates, along with the addition of 3.0% and 6.0% duck egg white jelly (B^3^ and B^6^ groups), the pH of the raw meat paste significantly exceeded that of the control group and group A^0^, both of which lacked duck egg white jelly (*p* < 0.05).

Furthermore, in group C^6^, where 6.0% duck egg white jelly and 0.2% phosphate were added to the raw meat paste, the pH significantly surpassed that of the other treatment groups (*p* < 0.05). These results suggest that both phosphate and duck egg white jelly increased the pH of the raw meat pastes, with the addition of duck egg white jelly resulting in a particularly significant pH increase (*p* < 0.05). Due to its alkaline pH of approximately 11.2, duck egg white jelly notably raised the pH of the raw meat pastes, with an effect that was slightly stronger than that of phosphate. Unlike other alkaline additives, such as eggshell powder, oyster shell powder, or sodium bicarbonate, which solely contribute ions to adjust pH, duck egg white jelly also contains proteins that may indirectly enhance stability by interacting with meat proteins and forming structural bonds. When minced and mixed with raw meat using a bowl cutter, the alkaline duck egg white jelly dispersed evenly and adhered to the meat paste. Furthermore, the inclusion of egg white jelly, which contains hydroxyl and salt ions, not only elevated the pH and ion concentration of the raw meat pastes but also facilitated the release of salt-soluble proteins from the meat and unfolded protein structures. As a result, these changes influenced the emulsion stability of the meat pastes, enhancing their ability to maintain a stable emulsion. This effect aligns with findings from previous studies, which have shown that alkaline additives, such as sodium bicarbonate, can enhance protein solubility and stability by altering ionic interactions within meat emulsions [15]. Thangavelu et al. [30] proposed that alkaline ingredients have the potential to substitute phosphates and improve the pH and water-holding capacity of raw meat pastes. However, the protein-rich nature of duck egg white jelly provides additional functional benefits, as it not only adjusts the pH but also enhances emulsion stability through improved protein interactions, potentially offering advantages over many other alkaline ingredients. This added functionality could significantly improve the quality of emulsified meat products. Based on this premise, it is plausible to consider that alkaline duck egg white jelly may have the ability to partially replace phosphates.

**Figure 2:**
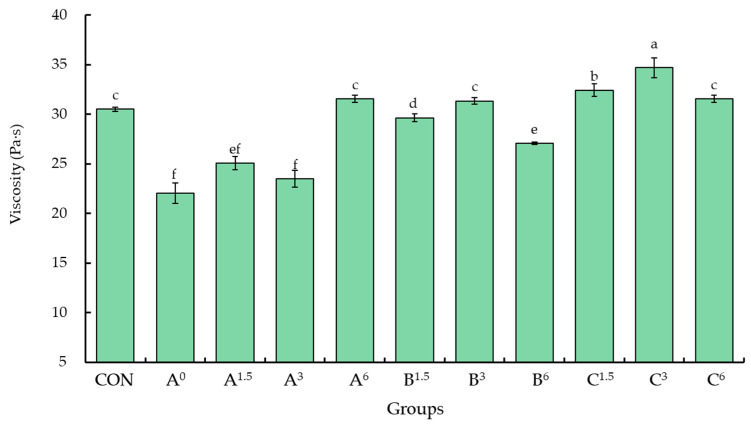
Effects of duck egg white jelly on the viscosity of raw meat pastes. Raw meat pastes were tested under three different treatments: A (without phosphate), B (with 0.1% phosphate), and C (with 0.2% phosphate). The superscript numbers indicate the percentage of duck egg white jelly added. Data are presented as means ± SEM (*n* = 3). Bars with different letters denote significant differences among groups (*p* < 0.05). CON (control) represents a commercial meatball formula containing 0.2% phosphate without duck egg white jelly.

The raw meat paste of group A^0^, which did not contain phosphate or duck egg white jelly, exhibited the lowest viscosity (Figure 2). As a result, the continuous phase of the meat emulsion displayed increased fluidity, leading to unstable emulsification and the occurrence of various defects, such as fat or grease accumulation in the emulsified products. Therefore, in the production process of emulsified meatballs, incorporating highly concentrated salt ions or phosphate can facilitate the release of salt-soluble proteins, enhance the viscosity and cohesiveness of the raw meat paste, and simultaneously maintain the stability and dispersion of fat in the continuous phase. The results of this study demonstrated that the addition of phosphate increased the viscosity of the meat paste. Among the groups containing 1.5% duck egg white jelly, C^1.5^ exhibited the highest viscosity, followed by B^1.5^, with intermediate viscosity, and A^1.5^, with the lowest viscosity. Similarly, within the groups containing 3.0% duck egg white jelly, C^3^ displayed the highest viscosity, followed by B^3^, with intermediate viscosity, and A^3^, with the lowest viscosity. Notably, in group C^3^, which contained 3.0% duck egg white jelly, the highest viscosity (*p* < 0.05) was recorded. However, there was no significant difference (*p* > 0.05) in the viscosity of the raw meat pastes of group A^6^ (no phosphate), group B^3^ (0.1% phosphate), or the control group (0.2% phosphate). Knipe et al. [31] reported that adding phosphate significantly increased the viscosity of raw meat pastes and improved emulsification stability [32]. According to Aktaş and Genccelep [33], raw meat pastes with higher viscosity tend to have greater cohesiveness and are more resistant to separation and breakdown during boiling. The results of the current study demonstrate that, in general, increasing the amount of phosphate or duck egg white jelly added to raw meat pastes leads to an increase in viscosity.

However, an interesting observation was made, that regardless of whether 0.1% or 0.2% phosphate was added, the viscosity of raw meat pastes decreased when 6% duck egg white jelly was included. One possible reason is that the excessive addition of duck egg white jelly may have increased the moisture content of the raw meat pastes, resulting in reduced viscosity. The raw meat paste without phosphate exhibited lower viscosity and decreased stability. The amount of added duck egg white jelly was increased to 6% to achieve a viscosity level comparable to the control group. The results also indicated that the changes in pH of the raw meat pastes were similar to those in viscosity. In other words, the raw meat pastes with a higher pH exhibited higher viscosity. Due to duck egg white jelly being an alkali-induced protein gel, it contains unfolded ovalbumin and other proteins that can bond or interact with other meat proteins, enhancing the viscosity and emulsifying capacity of raw meat pastes. In this study, the proteins in the meat combined with alkaline duck egg white jelly, causing the unfolding of protein structures and the exposure of different functional groups. The side-chain groups between adjacent protein molecules subsequently formed covalent and noncovalent bonds, enhancing their resistance. Additionally, the gel-forming aggregate bonded with water molecules, resulting in an increased degree of viscosity and the formation of a densely organized network structure within the raw meat pastes [34].

### 3.2. Effects of Alkaline Duck Egg White Jelly on the Texture Profile and Water-Holding Capacity of Emulsified Meat Snacks

According to Yapar et al. [35], increasing the viscosity of raw meat pastes can enhance the elasticity of cooked emulsified meat products and improve their texture profile. To examine the effect of duck egg white jelly on the texture of emulsified meatballs, differently treated raw meat pastes were stuffed into sausage casings and boiled until they reached their desired shape. Subsequently, a texture analysis was conducted. The emulsified meat snack samples from groups with more viscous raw meat pastes exhibited superior textural properties, including hardness, cohesiveness, gumminess, chewiness, and resilience (Table 2). Despite the reduction in viscosity caused by adding 6% duck egg white, it significantly enhanced the texture profile of the cooked samples. Particularly, group C^6^, which included 0.2% phosphate and 6% duck egg white jelly, displayed superior texture compared to the control group, which did not contain duck egg white jelly (*p* < 0.05).

Moreover, the texture profile of group C^6^ surpassed groups C^1.5^ and C^3^, which contained 1.5% and 3% duck egg white jelly, respectively. Regarding springiness, groups B and C demonstrated superior springiness compared to group A and the control group. These findings confirm that adding phosphate and alkaline duck egg white jelly improved the physical properties of emulsified meat snacks, including springiness and chewiness. Zheng et al. [27] reported that when duck egg white jelly was heated to 80 °C, the protein chains became stronger and formed a denser network structure. In the current study, after boiling the raw meat pastes containing evenly mixed and interlinked duck egg white proteins and meat proteins, the protein network structure became denser, and the protein chains became stronger, resulting in increased springiness and chewiness in the final meat product.

Phosphates are additives used in emulsified meat products to reduce weight loss and enhance texture and sensory properties. During the production process, the addition of phosphates facilitates the release of salt-soluble myofibrillar proteins from lean meat, which stabilizes the protein–water binding state and improves water-holding capacity [7,36]. High-quality emulsified meat products typically exhibit minimal shrinkage and dehydration during cooking. Therefore, cooking loss is an important indicator for evaluating the quality of emulsified meat products, in addition to water-holding capacity [37]. Various factors, such as cooking time, cooking temperature, cooking method, fat content, and sausage casing, can influence the cooking loss of emulsified meat products [38,39,40,41,42]. Goemaere et al. [25] suggested that chicken egg white powder can be used as a phosphate substitute in emulsified meat products, as it enhances stable emulsification, improves water-holding capacity, reduces cooking loss, and increases post-cooking hardness.

**Figure 3:**
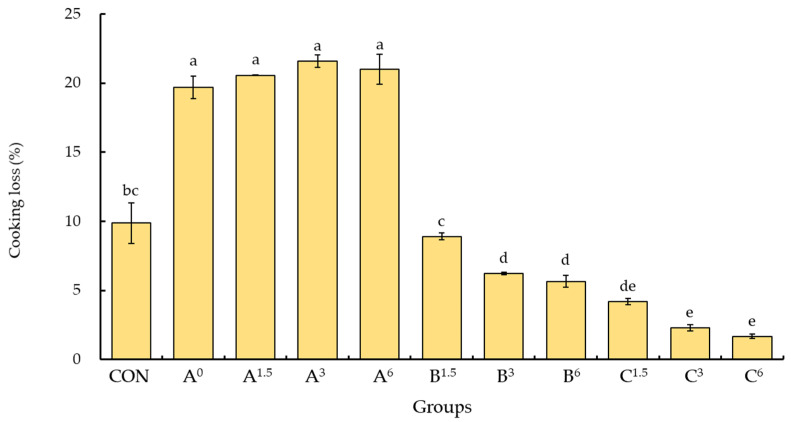
Effects of duck egg white jelly on the cooking loss of emulsified meat snacks. The cooking loss of emulsified meat snacks was assessed under three different treatments: A (no phosphate), B (0.1% phosphate), and C (0.2% phosphate). Superscript numbers indicate the percentage of duck egg white jelly added. Data are presented as means ± SEM (*n* = 3). Bars with different letters denote significant differences among groups (*p* < 0.05). CON (control) represents a commercial meatball formula containing 0.2% phosphate without duck egg white jelly.

However, adding liquid egg white may result in foam formation during mixing with lean meat and fat or an uneven distribution of egg white after heating, which can negatively impact the structural integrity, texture, and succulence of cooked emulsified meat products. Therefore, this study aimed to investigate the effect of duck egg white jelly on the cooking loss of emulsified meatballs. The cooking loss of group A^0^, which did not contain phosphate or duck egg white jelly, was approximately 19.7%, significantly higher than that of the control group (9.9%, *p* < 0.05; Figure 3). This indicates that the raw meat paste without phosphate lost over twice the amount of moisture compared to the control when boiled. Groups A^1.5^, A^3^, and A^6^, which only contained duck egg white jelly, had significantly higher cooking losses compared to groups B and C and the control group (*p* < 0.05). These results highlight the crucial role of phosphate in water retention in emulsified meat snacks. While the duck egg white jelly did not directly increase the water-holding capacity of the meat products, the cooking losses of groups B and C, which included both phosphate and duck egg white jelly, were significantly lower than that of the control group (*p* < 0.05, Figure 3).

Furthermore, the cooking loss decreased further with the additional inclusion of duck egg white jelly, confirming that the addition of alkaline duck egg white jelly, in combination with phosphate, effectively increased the water-holding capacity of the emulsified meatballs and influenced their structural stability and texture. To assess the water-holding capacity of emulsified meatballs during storage, the purge loss of the samples was analyzed after freezing for 0, 7, 14, 21, and 28 days (Table 3). The purge loss of group A, without phosphate, significantly exceeded that of the control group (*p* < 0.05). However, no significant difference in purge loss was observed between the other groups and the control groups on day 0 (*p* > 0.05). The results also showed increased purge loss for each treatment group, with a longer freezing duration. Specifically, during frozen storage, group A^0^, lacking phosphate or duck egg white jelly, exhibited pronounced purge loss, while groups B and C, containing both phosphate and alkaline egg white jelly, displayed a comparatively slower increase in purge loss. Overall, the water-holding capacity of emulsified meatballs showed a correlation with the amount of phosphate and duck egg white jelly used. Incorporating phosphate and duck egg white jelly effectively controlled the purge loss of emulsified meatballs and enhanced their textural properties. Thus, duck egg white jelly has the potential to enhance the quality of emulsified meat snacks while partially replacing phosphate additives.

### 3.3. Effects of Alkaline Duck Egg White Jelly on the Color and Sensory Properties of Emulsified Meatballs

Meat color is primarily determined by myoglobin, a water-soluble protein containing a central iron atom. The redox state of iron ions and their interaction with compounds such as oxygen or nitric oxide influence color changes in meat [43]. Additionally, changes in meat pH affect the quantity and properties of protein charges, altering the spacing and structure between meat fibers, which impact light reflection and absorption, ultimately affecting the visual assessment of meat color [43,44]. In this study, as the concentration of duck egg white jelly increased, the raw meat pastes observed a noticeable color change from pink to gray. This color change is likely attributed to the alkaline nature of duck egg white jelly, which raises the pH and oxidizes myoglobin. These findings confirm the significant role of pH in determining the color of raw meat pastes. After heating the raw meat pastes at 80 °C for 15 min, the denaturation of myoglobin and oxidation of iron ions resulted in the meat appearing light gray. Since visual observation alone cannot accurately discern these color differences, a color checker was employed to digitize the colors of the boiled emulsified meat snack samples in three-dimensional space, with L* representing brightness, a* representing redness, and b* representing yellowness (Table 4). Group A^6^ had the highest brightness (L*) and whiteness (*p* < 0.05), with no significant difference observed among the other groups.

Furthermore, the redness (a*) of group A^0^, which did not contain phosphate or duck egg white jelly, significantly exceeded that of the other groups (*p* < 0.05). However, emulsified meatballs containing 6.0% duck egg white jelly exhibited significantly lower redness (a*) compared to those with lower concentrations of duck egg white jelly or no duck egg white jelly at all (*p* < 0.05). These results indicate a higher degree of greenness, consistent with the visual observation of the raw meat paste color. The addition of phosphate and duck egg white jelly to the raw meat pastes increased their pH, affecting the protein chain’s network structure, water-holding capacity, and oxidation rate of iron ions in myoglobin. Upon heat treatment, myoglobin undergoes denaturation, leading to the increased oxidation of iron ions. This restricts protein chain contraction and dehydration, resulting in higher brightness and reduced redness in the color analysis.

Regarding yellowness, emulsified meat snacks with phosphate and 3% or more duck egg white jelly exhibited a higher level of yellowness. This phenomenon can be attributed to the significant increase in yellowness (b*) of duck egg white jelly when heated to 70 °C or higher [33]. However, after adding duck egg white jelly, the yellowness of group A, which did not contain phosphate, remained the same. Therefore, further research is required to determine whether the components of phosphate and duck egg white jelly are related to coloring substances.

To assess the impact of duck egg white jelly and phosphate on consumer acceptability and sensory scores of emulsified meat snacks, 11 different types of meatballs were compared and evaluated in this study. The sensory scores for color did not show significant differences, ranging from approximately 5.06 to 5.50 (*p* > 0.05) (Table 5). Incorporating an appropriate amount of duck egg white jelly and achieving a suitable reduction in phosphates resulted in improved scores for texture, flavor, and overall acceptability of the emulsified meatball samples. Particularly, group B^3^ exhibited superior texture and overall consumer acceptance compared to group A and the control group (*p* < 0.05) (Table 1 and Table 4). The panelists described the emulsified meatballs in group A^0^, which lacked both phosphate and duck egg white jelly, as loose and greasy. However, including duck egg white jelly significantly enhanced the texture and flavor of the emulsified meatballs, leading to improved sensory attributes. Overall, the emulsified meatballs containing duck egg white jelly received positive feedback and were well-received by the panelists. Meat snacks in group B, containing 50% less phosphate than the normal amount, achieved sensory scores of 6 or higher for attributes such as texture, flavor, and overall acceptance. To the authors’ knowledge, this study represents the successful integration of duck egg white jelly into meat products, producing emulsified meatballs containing duck egg white that consumers enjoy.

## 4. Conclusions

Duck egg white jelly is a versatile ingredient for both hot and cold dishes. It can be effectively incorporated into a blend of lean meat and fat to create emulsified meat snacks, reducing the need for added phosphate. Among the tested formulations, those containing 3.0% or 6.0% duck egg white jelly with 0.1% phosphate emerged as the most promising alternatives, demonstrating superior technological and sensory attributes. The addition of duck egg white jelly improves the raw meat paste’s viscosity, stabilizing the meat emulsion’s continuous phase. These emulsified meat snacks exhibit outstanding water-holding capacity and minimal cooking loss even after boiling. Furthermore, including duck egg white enhances the emulsified meatballs’ springiness and chewiness. This study successfully demonstrates the integration of duck egg white jelly with meat, leading to the development of specialized emulsified meat snacks. Since duck egg white is a protein-rich agricultural by-product, alkaline egg white jelly offers a promising opportunity to create innovative low-phosphate emulsified meat products with distinct textures and enhanced nutritional value.

## Figures and Tables

**Table 1:** Formulations of emulsified meat snacks with varying levels of phosphate and duck egg white jelly.

	Raw Meat Materials (100 kg)		Additives and Seasonings *					
Group	Lean Pork Meat (kg)	Back Fat (kg)	Phosphate (%)	Duck Egg White Jelly (%)	Salt (%)	Sugar (%)	Garlic Powder (%)	White Pepper Powder (%)
CON	75	25	0.2	0	1.3	2.0	0.2	0.2
A^0^	75	25	0	0	1.3	2.0	0.2	0.2
A^1.5^	75	25	0	1.5	1.3	2.0	0.2	0.2
A^3^	75	25	0	3.0	1.3	2.0	0.2	0.2
A^6^	75	25	0	6.0	1.3	2.0	0.2	0.2
B^1.5^	75	25	0.1	1.5	1.3	2.0	0.2	0.2
B^3^	75	25	0.1	3.0	1.3	2.0	0.2	0.2
B^6^	75	25	0.1	6.0	1.3	2.0	0.2	0.2
C^1.5^	75	25	0.2	1.5	1.3	2.0	0.2	0.2
C^3^	75	25	0.2	3.0	1.3	2.0	0.2	0.2
C^6^	75	25	0.2	6.0	1.3	2.0	0.2	0.2

Lean pork meat from the hind leg of pigs and back fat were mixed in a 3:1 ratio (75:25) to prepare emulsified meat snacks. * The percentage of additives and seasonings in the formulations was calculated based on the total combined weight of the lean pork meat and back fat (100%). CON: control group, based on a commercial meatball formula containing 0.2% phosphate and no duck egg white jelly. A: emulsified meat snacks without phosphate. B: emulsified meat snacks with 0.1% phosphate. C: emulsified meat snacks with 0.2% phosphate. Superscripts indicate the percentage of duck egg white jelly added to the respective formulations (e.g., 1.5%, 3.0%, 6.0%). All formulations include identical amounts of sugar, salt, garlic powder, and other seasonings to maintain consistent flavor and texture profiles.

**Table 2:** Texture profile analysis of emulsified meat snacks with varying phosphate and duck egg white jelly content.

Groups	Hardness (N)	Springiness	Cohesiveness	Gumminess (N)	Chewiness (N·mm)	Resilience
CON	99.04 ± 1.15 ^c^	0.78 ± 0.08 ^cd^	0.64 ± 0.01 ^e^	62.97 ± 1.32 ^b^	49.18 ± 5.68 ^c^	0.26 ± 0.00 ^d^
A^0^	63.22 ± 0.65 ^g^	0.76 ± 0.01 ^d^	0.43 ± 0.01 ^g^	27.32 ± 0.89 ^e^	20.79 ± 0.89 ^de^	0.13 ± 0.01 ^g^
A^1.5^	50.14 ± 0.63 ^i^	0.77 ± 0.01 ^cd^	0.48 ± 0.01 ^f^	24.12 ± 0.35 ^e^	18.57 ± 0.52 ^e^	0.16 ± 0.01 ^f^
A^3^	56.23 ± 0.18 ^h^	0.79 ± 0.03 ^cd^	0.44 ± 0.03 ^g^	24.93 ± 1.51 ^e^	19.68 ± 0.87 ^de^	0.15 ± 0.02 ^fg^
A^6^	64.50 ± 1.05 ^g^	0.78 ± 0.01 ^cd^	0.49 ± 0.01 ^f^	31.80 ± 0.94 ^d^	24.79 ± 0.96 ^d^	0.18 ± 0.01 ^e^
B^1.5^	87.74 ± 0.51 ^e^	0.93 ± 0.01 ^a^	0.68 ± 0.01 ^cd^	58.06 ± 0.21 ^c^	53.92 ± 0.70 ^bc^	0.29 ± 0.00 ^bc^
B^3^	78.96 ± 0.63 ^f^	0.91 ± 0.01 ^ab^	0.73 ± 0.01 ^a^	56.21 ± 0.61 ^c^	51.84 ± 0.16 ^bc^	0.33 ± 0.00 ^a^
B^6^	104.12 ± 2.56 ^a^	0.85 ± 0.03 ^abcd^	0.72 ± 0.00 ^ab^	65.84 ± 2.81 ^b^	57.59 ± 1.34 ^ab^	0.31 ± 0.00 ^b^
C^1.5^	91.79 ± 0.62 ^d^	0.84 ± 0.04 ^abcd^	0.69 ± 0.02 ^bc^	63.67 ± 1.58 ^b^	53.63 ± 3.14 ^bc^	0.30 ± 0.01 ^bc^
C^3^	99.46 ± 1.69 ^bc^	0.86 ± 0.02 ^abc^	0.65 ± 0.01 ^de^	64.62 ± 0.90 ^b^	55.65 ± 0.95 ^ab^	0.29 ± 0.00 ^c^
C^6^	102.80 ± 1.65 ^ab^	0.83 ± 0.01 ^bcd^	0.71 ± 0.00 ^abc^	73.16 ± 1.15 ^a^	60.52 ± 0.56 ^a^	0.34 ± 0.00 ^a^

CON = control (commercial meatball formula with 0.2% phosphate and no duck egg white jelly added). A: emulsified meat snacks without phosphate; B: emulsified meat snacks with 0.1% phosphate; C: emulsified meat snacks with 0.2% phosphate. Superscripts denote the percentage of duck egg white jelly. Data are presented as means ± SEM (*n* = 3). Data bars with different letters indicate significant differences (*p* < 0.05) between the groups.

**Table 3:** Effects of duck egg white jelly on the purge loss of emulsified meat snacks.

	Purge Loss (%)				
Groups	0 Day	7 Day	14 Day	21 Day	28 Day
CON	1.09 ± 0.07 ^b^	1.24 ± 0.02 ^b^	1.29 ± 0.09 ^cd^	2.09 ± 0.09 ^c^	2.16 ± 0.14 ^cd^
A^0^	1.81 ± 0.08 ^a^	2.23 ± 0.03 ^a^	3.11 ± 0.05 ^a^	3.82 ± 0.65 ^a^	3.94 ± 0.22 ^a^
A^1.5^	1.71 ± 0.02 ^a^	2.19 ± 0.08 ^a^	2.28 ± 0.01 ^b^	2.38 ± 0.01 ^b^	2.40 ± 0.01 ^c^
A^3^	1.81 ± 0.02 ^a^	2.16 ± 0.02 ^a^	2.23 ± 0.09 ^b^	2.34 ± 0.19 ^b^	2.35 ± 0.09 ^c^
A^6^	1.89 ± 0.01 ^a^	2.17 ± 0.02 ^a^	2.35 ± 0.05 ^b^	2.48 ± 0.05 ^b^	2.82 ± 0.65 ^b^
B^1.5^	1.18 ± 0.05 ^b^	1.23 ± 0.03 ^b^	1.35 ± 0.07 ^c^	1.89 ± 0.06 ^cd^	2.23 ± 0.28 ^cd^
B^3^	0.85 ± 0.10 ^c^	0.94 ± 0.17 ^d^	1.07 ± 0.06 ^e^	1.57 ± 0.36 ^e^	1.97 ± 0.06 ^d^
B^6^	1.24 ± 0.14 ^b^	1.27 ± 0.09 ^b^	1.44 ± 0.03 ^c^	1.84 ± 0.37 ^d^	2.34 ± 0.37 ^c^
C^1.5^	1.06 ± 0.04 ^b^	1.11 ± 0.02 ^c^	1.20 ± 0.02 ^d^	1.80 ± 0.02 ^d^	1.90 ± 0.12 ^d^
C^3^	1.14 ± 0.04 ^b^	1.17 ± 0.06 ^c^	1.24 ± 0.09 ^cd^	1.84 ± 0.09 ^d^	1.99 ± 0.09 ^d^
C^6^	1.08 ± 0.09 ^b^	1.15 ± 0.03 ^c^	1.22 ± 0.01 ^cd^	1.82 ± 0.01 ^d^	1.92 ± 0.01 ^d^

CON = control (commercial meatball formula with 0.2% phosphate and no duck egg white jelly added). A: emulsified meat snacks without phosphate; B: emulsified meat snacks with 0.1% phosphate; C: emulsified meat snacks with 0.2% phosphate. Superscripts denote the percentage of duck egg white jelly. Data are presented as means ± SEM (*n* = 3). Data bars with different letters indicate significant differences (*p* < 0.05) between the groups.

**Table 4:** Changes in L*, a*, b* values and the whiteness of emulsified meat snacks with varying levels of phosphate and egg white jelly content.

Groups	L*	a*	b*	Whiteness
CON	75.34 ± 0.50 ^cd^	1.83 ± 0.25 ^abc^	14.86 ± 0.40 ^cde^	71.14 ± 0.42 ^cd^
A^0^	76.94 ± 0.51 ^abc^	2.15 ± 0.16 ^a^	14.97 ± 0.29 ^cde^	72.42 ± 0.54 ^abc^
A^1.5^	76.26 ± 1.42 ^bc^	1.41 ± 0.20 ^cde^	14.47 ± 0.18 ^e^	72.14 ± 1.15 ^bc^
A^3^	76.34 ± 0.23 ^bc^	1.91 ± 0.06 ^ab^	14.94 ± 0.05 ^cde^	71.96 ± 0.22 ^bc^
A^6^	78.25 ± 0.35 ^a^	1.24 ± 0.12 ^def^	14.77 ± 0.19 ^cde^	73.68 ± 0.40 ^a^
B^1.5^	75.72 ± 0.51 ^bcd^	1.05 ± 0.04 ^ef^	14.92 ± 0.16 ^cde^	71.48 ± 0.13 ^bcd^
B^3^	75.83 ± 0.21 ^bcd^	1.58 ± 0.03 ^bcd^	15.93 ± 0.19 ^a^	71.01 ± 0.23 ^cd^
B^6^	76.20 ± 0.43 ^bc^	0.99 ± 0.02 ^f^	15.69 ± 0.15 ^ab^	71.48 ± 0.43 ^bcd^
C^1.5^	77.35 ± 0.48 ^ab^	1.34 ± 0.22 ^def^	14.72 ± 0.24 ^de^	72.95 ± 0.52 ^ab^
C^3^	74.36 ± 0.10 ^d^	1.93 ± 0.06 ^ab^	15.23 ± 0.13 ^bcd^	70.11 ± 0.13 ^d^
C^6^	75.74 ± 0.11 ^bcd^	1.21 ± 0.13 ^def^	15.40 ± 0.36 ^abc^	71.24 ± 0.29 ^cd^

CON = control (commercial meatball formula with 0.2% phosphate and no duck egg white jelly added). A: emulsified meat snacks without phosphate; B: emulsified meat snacks with 0.1% phosphate; C: emulsified meat snacks with 0.2% phosphate. Superscripts denote the percentage of duck egg white jelly. Data are presented as means ± SEM (*n* = 3). Data bars with different letters indicate significant differences (*p* < 0.05) between the groups.

**Table 5:** Effects of duck egg white jelly on the sensory properties of cooked emulsified meat snacks.

Groups	Color	Texture	Flavor	Total Acceptability
CON	5.22 ± 0.21	6.31 ± 0.19 ^b^	6.31 ± 0.22 ^ab^	6.33 ± 0.22 ^b^
A^0^	5.14 ± 0.22	4.31 ± 0.29 ^d^	4.61 ± 0.35 ^d^	4.56 ± 0.32 ^d^
A^1.5^	5.11 ± 0.22	4.75 ± 0.27 ^cd^	5.00 ± 0.36 ^cd^	5.00 ± 0.34 ^cd^
A^3^	5.06 ± 0.24	5.28 ± 0.28 ^c^	5.81 ± 0.31 ^b^	5.36 ± 0.29 ^c^
A^6^	5.08 ± 0.22	4.56 ± 0.31 ^cd^	5.17 ± 0.33 ^bc^	4.97 ± 0.30 ^cd^
B^1.5^	5.24 ± 0.20	6.78 ± 0.17 ^a^	6.66 ± 0.24 ^a^	6.77 ± 0.25 ^ab^
B^3^	5.36 ± 0.19	7.06 ± 0.20 ^a^	6.54 ± 0.23 ^a^	6.98 ± 0.27 ^a^
B^6^	5.19 ± 0.26	6.83 ± 0.29 ^a^	6.55 ± 0.28 ^a^	6.56 ± 0.21 ^ab^
C^1.5^	5.50 ± 0.25	6.81 ± 0.22 ^a^	6.67 ± 0.23 ^a^	6.83 ± 0.22 ^ab^
C^3^	5.33 ± 0.22	6.81 ± 0.21 ^a^	6.53 ± 0.21 ^a^	6.67 ± 0.24 ^ab^
C^6^	5.33 ± 0.24	6.61 ± 0.24 ^ab^	6.22 ± 0.28 ^ab^	6.39 ± 0.28 ^b^

CON = control (commercial meatball formula with 0.2% phosphate and no duck egg white jelly added). A: emulsified meat snacks without phosphate; B: emulsified meat snacks with 0.1% phosphate; C: emulsified meat snacks with 0.2% phosphate. Superscripts denote the percentage of duck egg white jelly. Data are presented as means ± SEM (*n* = 35). Data bars with different letters indicate significant differences (*p* < 0.05) between the groups.

## Data Availability

All data supporting the findings of this study are contained within the article; further inquiries can be directed to the corresponding author.
